# Autoregulation of Transcription and Translation: A Qualitative Analysis

**DOI:** 10.1007/s11538-023-01143-6

**Published:** 2023-05-26

**Authors:** Philip J. Murray

**Affiliations:** University of Dundee, Dundee, UK

**Keywords:** Translation, Molecular oscillator, Relaxation oscillator, Transcription, Notch signalling

## Abstract

The regulation of both mRNA transcription and translation by down-stream gene products allows for a range of rich dynamical behaviours (e.g. homeostatic, oscillatory, excitability and intermittent solutions). Here, qualitative analysis is applied to an existing model of a gene regulatory network in which a protein dimer inhibits its own transcription and upregulates its own translation rate. It is demonstrated that the model possesses a unique steady state, conditions are derived under which limit cycle solutions arise and estimates are provided for the oscillator period in the limiting case of a relaxation oscillator. The analysis demonstrates that oscillations can arise only if mRNA is more stable than protein and the effect of nonlinear translation inhibition is sufficiently strong. Moreover, it is shown that the oscillation period can vary non-monotonically with transcription rate. Thus the proposed framework can provide an explanation for observed species-specific dependency of segmentation clock period on Notch signalling activity. Finally, this study facilitates the application of the proposed model to more general biological settings where post transcriptional regulation effects are likely important.

## Introduction

The design principles that underpin oscillations in biological systems are naturally described using mathematical approaches (Alon 2019; Winfree 2001; Novák and Tyson 2008; Tyson and Novák 2010). There are now numerous well established models across a range of cellular oscillators [e.g. cell cycle, circadian cycle, cardiac cycle, glycolysis (Sel’Kov 1968), NF$\kappa$b (Gonze and Abou-Jaoudé 2013), p. 53, (Geva-Zatorsky et al. 2006)].

A conserved principle of the Hes/Her oscillator, now known to be present in many different cell types (Kageyama et al. 2007), is that dimerised members of the basic Helix-loop-helix family of transcription factors (e.g. Hes7, Hes1, Her7) inhibit their own transcription and therefore provide a negative feedback loop. The Notch signalling pathway, which plays a crucial role in embryo development, tissue homeostasis (van Es et al. 2005) and cancer (Mollen et al. 2018; Allenspach et al. 2002; Siebel and Lendahl 2017), can activate the transcription of Hes/Her genes. During canonical *in trans* Notch signalling, a Notch ligand in a signalling cell activates a Notch receptor in a neighbour, resulting in the release of the Notch intracellular domain (NICD) in the receiver, which regulates the transcription of Notch target genes. As at least in some biological contexts, such as the segmentation clock, Notch receptors are themselves a target of Notch signalling and levels of the Delta ligand can be regulated by Hes7 (Bone et al. 2014), the study of Notch signalling is a highly nonlinear problem.

Upon inclusion of time delays that represent processes such as transcription, splicing, transport and translation, it has been shown that negative feedback of transcription is sufficient to give rise to oscillations (Lewis 2003; Monk 2003). Moreover, it has been shown that the spatial diffusion of mRNA and protein is a sufficient mechanism to give rise to oscillations in a negative feedback system (Sturrock et al. 2011; Chaplain et al. 2015). Each of the above models makes the assumption that the translation of mRNA is linear, and thus unregulated.

Recent experimental observations challenge, at least in specific biological contexts, many existing models of the Notch signalling pathway. Oates and coworkers have demonstrated that when levels of Delta ligand are increased in presomitic mesoderm (PSM) cells, the tissue scale oscillator period decreases (Liao et al. 2016). Moreover, when levels of Notch signalling are reduced via treatment with the gamma secretase inhibitor DAPT, which blocks the release of NICD, the tissue scale oscillator period increases (Herrgen et al. 2010). Thus in the zebrafish embryo, the tissue-scale oscillator period appears to be anticorrelated with Notch signalling activity. In contrast, Dale and coworkers have demonstrated that when mouse and chick embryos are exposed to pharmacological treatments that increase levels of NICD, the tissue scale period increases (Wiedermann et al. 2015). Notably, a prediction of the delayed feedback models of the Her oscillator (Lewis 2003) is that the clock period has a strong dependence on the mRNA and protein half lives and time delays but not on transcription or translation rates (Lewis 2003).

Suggestions that mouse PSM tissue behaves like an excitable medium are also difficult to reconcile with delayed negative feedback models of the Notch signalling pathway. It has been identified that NICD is necessary for the oscillations of the segmentation clock in the presence of mechanosensitive Yap signalling (Hubaud et al. 2017). However, when Yap signalling is pharmacologically inhibited, oscillations could still proceed in the absence of Notch signalling. The presence of a Yap-signalling dependent threshold led the authors to conclude that the system under study behaved like an excitable medium. However, there is currently no molecular scale model of Hes7 dynamics that can account for such excitability.

In the Hes1 oscillator in mouse neural cells, the miRNA mir-9 has been identified (Bonev et al. 2012; Goodfellow et al. 2014) as a part of a double negative (i.e. positive) feedback loop in which mir-9 is under the same transcriptional control as the Hes1 gene but serves to inhibit translation. Together, these observations indicate that, at least in some specific biological contexts, the negative feedback model of the Notch signalling pathway is incomplete.

**Fig. 1:**
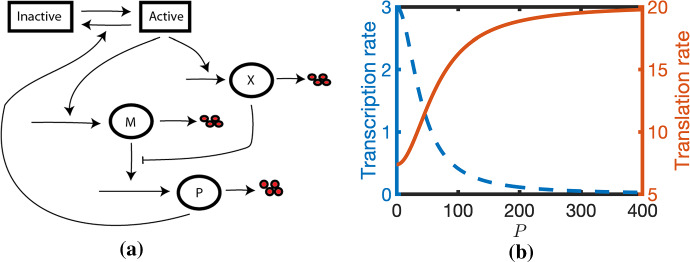
**a** A schematic illustration of the model. Active/Inactive denote transcriptional states of gene. mRNA is transcribed when the gene is active and degrades. Protein is translated from mRNA and degrades. **b** Translation and transcription rates plotted against protein levels, *P*

We recently developed an ordinary differential equation model that postulates that an intermediary, *X*, that is under the same transcriptional control as mRNA, M, inhibits translation [see Fig. 1a (Murray et al. 2021)]. Assuming quasi equilibrium for *X*, these assumptions introduce a positive feedback loop such that the translation rate increases sigmoidally with protein concentration (see Fig. 1b). Letting $M = M(t)$ and $P = P(t)$ represent the concentrations of mRNA and its corresponding protein at time *t*, respectively, the governing ODEs are

1 $$\begin{aligned} \frac{\textrm{d}M}{\textrm{d}t}&= \frac{k_1}{1+\left( \frac{P}{P_0}\right) ^2} - k_2 M, \nonumber \\ \frac{\textrm{d}P}{\textrm{d}t}&= M\left( k_3+\frac{k_4}{1+\dfrac{\alpha }{X_0}\frac{1}{\left( 1+ \left( \frac{P}{P_0}\right) ^2\right) }}\right) - k_5P, \end{aligned}$$

where $k_1$ is the maximal transcription rate, $P_0$ is the protein concentration at which transcription rate is half maximal, $k_2$ is the mRNA degradation rate, $k_3$ is the basal translation rate, $k_4$ is a translation rate that is inhibited by *X*, $\alpha$ is maximal level of *X* at steady state, $X_0$ is an IC50 constant for translational activation and $k_5$ is the protein degradation rate.

Using parameter values based on the zebrafish Her oscillator, it was shown, using numerical exploration, that Eq. (1) can possess excitable, homeostatic or oscillatory solutions (Murray et al. 2021). Using numerical continuation it was shown that Eq. (1) possess a subcritical Hopf bifurcation such that, in a particular region of parameter space, unstable limit cycle, stable limit cycle and stable steady state solutions coexist. In this case a stochastic implementation of the model is capable of exhibiting intermittent oscillations whereby noise switches the dynamics between a stable limit cycle and a stable steady state. Finally, it was shown that the oscillator period has, for the considered parameters, an inverse dependence on the transcription rate $k_1$. Hence the proposal that regulation of translation, as well as transcription, rates provides a minimal framework that yields phenomena consistent with recent experimental observations.

Whilst the previous work used numerical solutions to demonstrate interesting model behaviours, a qualitative analysis of the model behaviour is required in order that the model can be explored in more general biological contexts. Here this issue is addressed. The approach taken allows one to relate different Notch signalling behaviours in species-specific contexts (e.g. in which reaction rates may differ significantly). Parameter regimes are identified in which one expects to find different modes of behaviour (e.g. excitability, homeostasis, oscillations). Finally, an estimate is derived for the oscillator period and amplitude in the relaxation oscillator limit.

## Nondimensionalisation

Consider the dimensionless variables

$$\begin{aligned} m=\frac{M}{\tilde{M}}, \quad p=\frac{P}{\tilde{P}} \quad \text {and} \quad \tau =\frac{t}{\tilde{T}}. \end{aligned}$$

Letting

$$\begin{aligned} \tilde{M}= \frac{k_5 P_0}{k_3}\frac{\left( 1+\frac{\alpha }{X_0}\right) ^{\frac{1}{2}}}{\left( 1+\frac{k_4}{k_3}\right) }, \quad \tilde{P}=P_0\left( 1+\frac{\alpha }{X_0}\right) ^\frac{1}{2} \quad \text {and} \quad \tilde{T}=\frac{1}{k_5}, \end{aligned}$$

Equation (1) transforms to the nondimensional form

2 $$\begin{aligned} \frac{\textrm{d} m}{\textrm{d}\tau }&=\frac{\eta _1}{1+\frac{p^2}{\eta _2}} - \eta _3 m,\nonumber \\ \frac{\textrm{d} p}{\textrm{d}\tau }&=m\frac{(\eta _4+ p^2)}{1+p^2}-p, \end{aligned}$$

where

3 $$\begin{aligned} \eta _1&=\frac{k_1(k_3+k_4)}{k_5^2P_0\sqrt{1+\frac{\alpha }{X_0}}}, \quad \eta _2&=\frac{1}{1+\frac{\alpha }{X_0}}, \quad \eta _3&=\frac{k_2}{k_5},\quad \eta _4&=\frac{1+\frac{k_4}{k_3}+\frac{\alpha }{X_0}}{\left( 1+\frac{k_4}{k_3}\right) \left( 1+\frac{\alpha }{X_0}\right) }. \end{aligned}$$

Note that time has been nondimensionalised on the protein degradation timescale, the parameter $\eta _4$ represents the strength of the sigmoidal effect on translation rate and that $\eta _3$ is the ratio of mRNA to protein degradation rates. See Table 1 for typical values.

## Nullclines

**Fig. 2:**
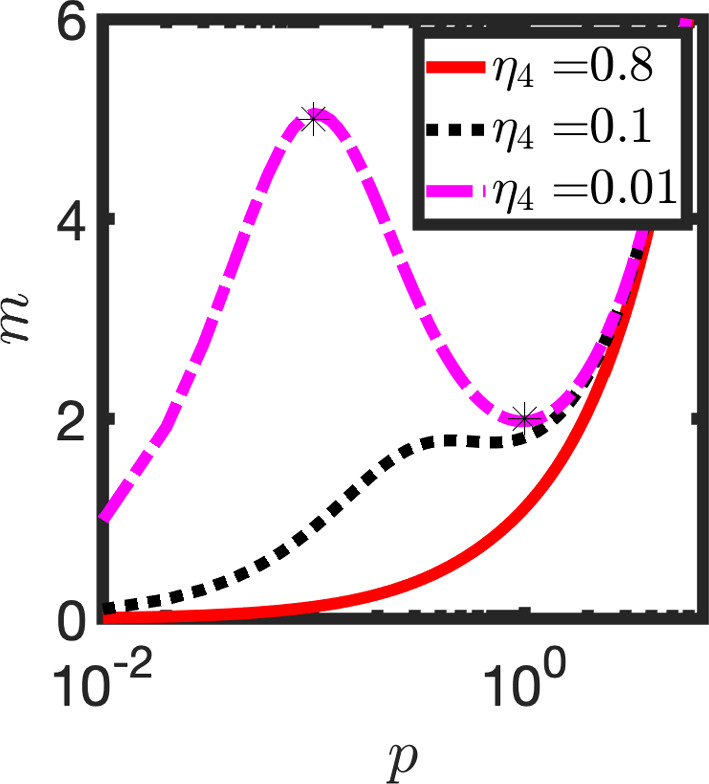
The *p* nullcline [see Eq. (4)] is plotted against *p* for different values of the parameter $\eta _4$. Markers denote coordinates of the extrema. Other parameters as in Table 1

The *p* nullcline, given by

4 $$\begin{aligned} {\bar{m}}_2(p)=\frac{p(1+p^2)}{\eta _4+p^2}, \end{aligned}$$

has two distinct real positive turning points if the condition

$$\begin{aligned} \eta _4<\frac{1}{9} \end{aligned}$$

holds (see Appendix A). The turning points occur at approximately

$$\begin{aligned} (p_1,m_1)=\left( {\sqrt{\eta _4}},\frac{1}{2\sqrt{\eta _4}}\right) , \end{aligned}$$

and

$$\begin{aligned} (p_2,m_2)=(1,2), \end{aligned}$$

(see Fig. 2). Note that the condition $\eta _4<1/9$ implies that $m_1>m_2$ and $p_1<p_2$. Hence $(p_1,m_1)$ is a local maximum and $(p_2,m_2)$ is a local minimum.

**Table 1:** A table of dimensionless parameter values

Parameter	Description	Value
$\eta _1$	mRNA transcription	0.76
$\eta _2$	Transcriptional inhibition IC50	0.008
$\eta _3$	mRNA degradation	0.02
$\eta _4$	Translation	0.01

The *m* nullcline, given by

5 $$\begin{aligned} {\bar{m}}_1(p)=\frac{\eta _1}{\eta _3}\frac{1}{1+\frac{p^2}{\eta _2}}, \end{aligned}$$

is monotonically decreasing for $p>0$ with an IC50 at $p=\sqrt{\eta _2}$ and local maximum of $\eta _1/\eta _3$. In order that nondimensional parameters correspond to positive dimensional parameters, the condition

$$\begin{aligned} \eta _2<\eta _4 \end{aligned}$$

must hold (see Appendix A). Hence the IC50 for transcriptional inhibition must be at least an order of magnitude less than the IC50 for the translational switch (which occurs at approximately $p=1$).

## Steady State Analysis

Suppose that $(m^*,p^*)$ is a steady state of Eq. (2). Upon elimination of $m^*$, $p^*$ satisfies the fifth order polynomial

6 $$\begin{aligned} h(p^*)=p^*{^5}+p^*{^3}(\eta _2+1)-p^*{^2} \frac{\eta _1\eta _2}{\eta _3}+\eta _2p^*-\frac{\eta _1\eta _2\eta _4}{\eta _3}=0. \end{aligned}$$

Recalling that $\eta _j>0 \ \forall \ j$, application of Descartes’ rule of signs implies that there are at most three real positive solutions of Eq. (6). Moreover, as

$$\begin{aligned} h(0)=-\frac{\eta _1\eta _2\eta _4}{\eta _3}<0 \quad \text {and} \quad h(p^*)\rightarrow \infty \quad \text {as} \quad p^*\rightarrow \infty , \end{aligned}$$

Equation (6) must have at least one real positive solution. Applying a graphical method (see Appendix B) it can be shown that, for biologically relevant parameter values, Eq. (6) possesses exactly one solution. This result precludes the possibility of bistability.

## Linear Stability Analysis

After substitution for the identity

$$\begin{aligned} m^*=\frac{p^*(1+{p^*}^2)}{\eta _4+{p^*}^2}, \end{aligned}$$

the Jacobian matrix of equations (2) takes the form

7 $$\begin{aligned} J= \left( \begin{array}{ll} -\eta _3 &{} -2\frac{\eta _1}{\eta _2}\dfrac{p}{\left( 1+\frac{p^2}{\eta _2}\right) ^2} \\ \dfrac{\eta _4+p^2}{1+p^2} &{} -\frac{p^4+p^2(3\eta _4-1)+\eta _4}{(\eta _4+p^2)(1+p^2)}\\ \end{array} \right) _{(m^*,p^*)}. \end{aligned}$$

Given that Eq. (3) possesses a unique steady state in the positive quadrant, the sign structure of the Jacobian matrix is given by

8 $$\begin{aligned} \left( \begin{array}{ll} -&{} -\\ + &{} \pm \\ \end{array} \right) . \end{aligned}$$

### Intersections on the Left and Right Branches are Linearly Stable

Negativity of the (2, 2) entry of the Jacobian matrix implies that

9 $$\begin{aligned} {p^*}^4+{p^*}^2(3\eta _4-1)+\eta _4>0, \end{aligned}$$

the left-hand side of which has previously been used to compute the turning points of the *p* nullcline (labelled as $p_1$ and $p_2$, see Eq. (19) in Appendix A). Hence when $p^*<p_1$ or $p^*>p_2$, such that the intersection between the *m* and *p* nullclines occurs on the left- or right-most branches of the *p* nullcline, respectively, the (2, 2) entry of the Jacobian matrix is negative and the steady state of Eq. (2) is therefore linearly stable.

#### The Steady State on the Central Branch of the *p* Nullcline is Conditionally Linearly Stable

The determinant of the Jacobian matrix is positive definite (see Appendix C). Hence the unique steady state of Eq. (2) is linearly unstable if and only if

10 $$\begin{aligned} \text {tr}{(J)}=-\eta _3-\frac{p^4+p^2(3\eta _4-1)+\eta _4}{(\eta _4+p^2)(1+p^2)} >0. \end{aligned}$$

This inequality can be expressed as

11 $$\begin{aligned} {p^*}^4 (1+\eta _3) - {p^*}^2 \left( 1-3\eta _4-\eta _3(1+\eta _4)\right) +\eta _4(1+\eta _3)<0, \end{aligned}$$

with the boundaries of the solution interval given by

12 $$\begin{aligned} p_{c}=\pm \left( \frac{1-3\eta _4-\eta _3(1+\eta _4)\pm \left( (1-3\eta _4-\eta _3(1+\eta _4))^2-4\eta _4(1+\eta _3)^2 \right) ^{\frac{1}{2}} }{2(1+\eta _3)} \right) ^{\frac{1}{2}}. \end{aligned}$$

For a real and positive solution interval it is therefore required that

$$\begin{aligned} 1-3\eta _4-\eta _3(1+\eta _4)>0 \implies \eta _3<\frac{1-3\eta _4}{1+\eta _4} \implies \eta _3<1, \end{aligned}$$

and

$$\begin{aligned} \left( (1-3\eta _4-\eta _3(1+\eta _4))^2-4\eta _4(1+\eta _3)^2 \right) >0, \end{aligned}$$

which can, upon rearrangement, be written as

$$\begin{aligned} \eta _4< \left( \frac{\eta _3-1}{3+\eta _3}\right) ^2. \end{aligned}$$

Considering the case where $\eta _3<1$, a necessary (but not sufficient) condition for instability of the steady state is

13 $$\begin{aligned} \eta _3<\frac{1-3\sqrt{\eta _4}}{1+\sqrt{\eta _4}}. \end{aligned}$$

In summary, when the conditions

14 $$\begin{aligned} \eta _2<\eta _4<\frac{1}{9}, \quad \eta _3<\frac{1-3\sqrt{\eta _4}}{1+\sqrt{\eta _4}} \end{aligned}$$

hold, there is always a real interval of $p^*$ within which $\text {tr}{(A)}$ is positive and the steady state is therefore linearly unstable. As $p^*$ is a monotonically increasing function of $\eta _1$ (see Appendix B) a corresponding interval of the parameter $\eta _1$ can always be found such that the unique steady state ($p^*$, $m^*$) is linearly unstable. This result implies that too little or too much basal transcription (i.e. $k_1$) will result in the disappearance of oscillatory solutions.

## Limit Cycle Solutions

A confined set can be defined for Eq. (2) (see Appendix D). Given the existence of a unique steady state, the Poincare Bendixson theorem can be applied in order to show that there is an interval of the parameter $\eta _1$ for which Eq. (2) have limit cycle solutions.

### Numerical Continuation

**Fig. 3:**
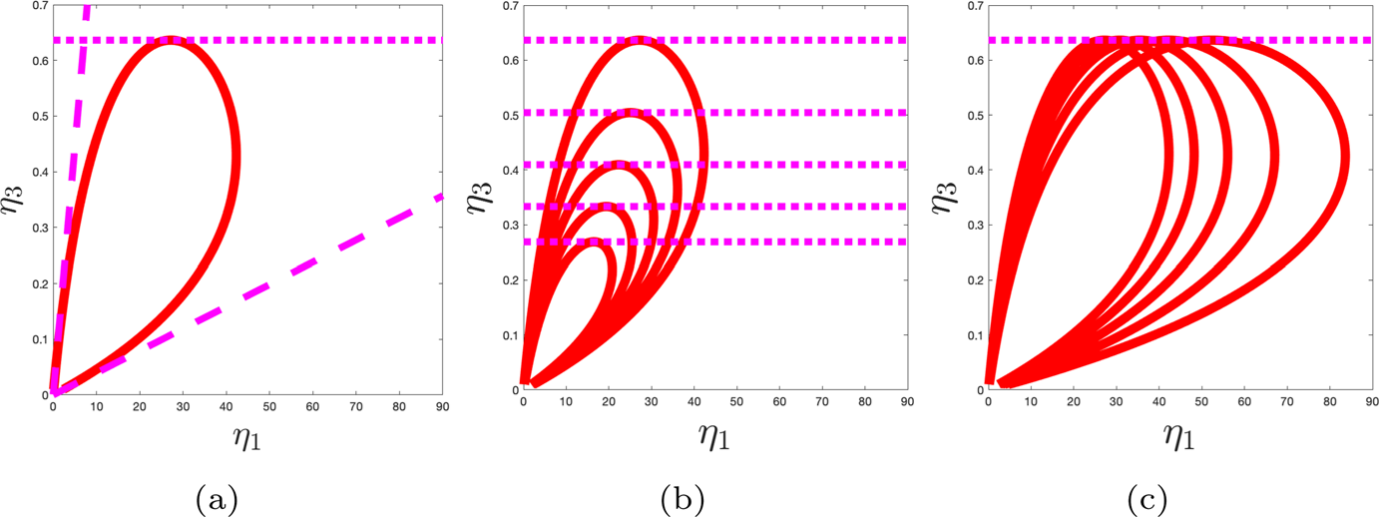
Planar projections of Hopf bifurcation surface. **a** A family of Hopf bifurcation points are represented by the red curve in the $\eta _1-\eta _3$ plane. The dotted horizontal line represents condition (14). Dashed lines represent equations (15). **b** Each loop in the $\eta _1-\eta _3$ plane represents a family of Hopf bifurcations at a different fixed value of the parameter $\eta _4$. **c** Each loop in the $\eta _1-\eta _3$ plane represents a family of Hopf bifurcations at a different fixed value of the parameter $\eta _2$. Parameter values as in Table 1 unless otherwise stated (Color figure online)

Numerical continuation was performed (Dankowicz and Schilder 2013) to confirm the presence of a family of Hopf bifurcations in the $\eta _1-\eta _3$ plane (see Fig. 3a). These numerical results indicate that, given that inequalities (14) hold, one can find an interval of the parameter $\eta _1$ in which there are limit cycle solutions. Note that the derived upper bound on $\eta _3$, given by inequality (13), is consistent with the upper bound estimated using continuation. Moreover, by imposing the conditions

$$\begin{aligned} m_2(p_1)>m_1(p_1) \quad \text {and} \quad m_2(p_2)<m_2(p_1), \end{aligned}$$

such that the nullclines intersect in the middle branch of the *p* nullcline, a necessary condition for limit cycle solutions is

15 $$\begin{aligned} \dfrac{1+\dfrac{\eta _4}{\eta _2}}{2\sqrt{\eta _4}}<\frac{\eta _1}{\eta _3}< 2\left( 1+\dfrac{1}{\eta _2}\right) . \end{aligned}$$

These bounds are presented in Fig. 3a. In Fig. 3b, c the Hopf bifurcation surface is projected onto the $\eta _1-\eta _3$ plane for different values of $\eta _4$ and $\eta _2$, respectively. Note that, as expected, the maximum value of $\eta _3$ for which oscillatory solutions are possible varies with the parameter $\eta _4$ (see Fig. 3b) but not $\eta _2$ (see Fig. 3c).

**Fig. 4:**
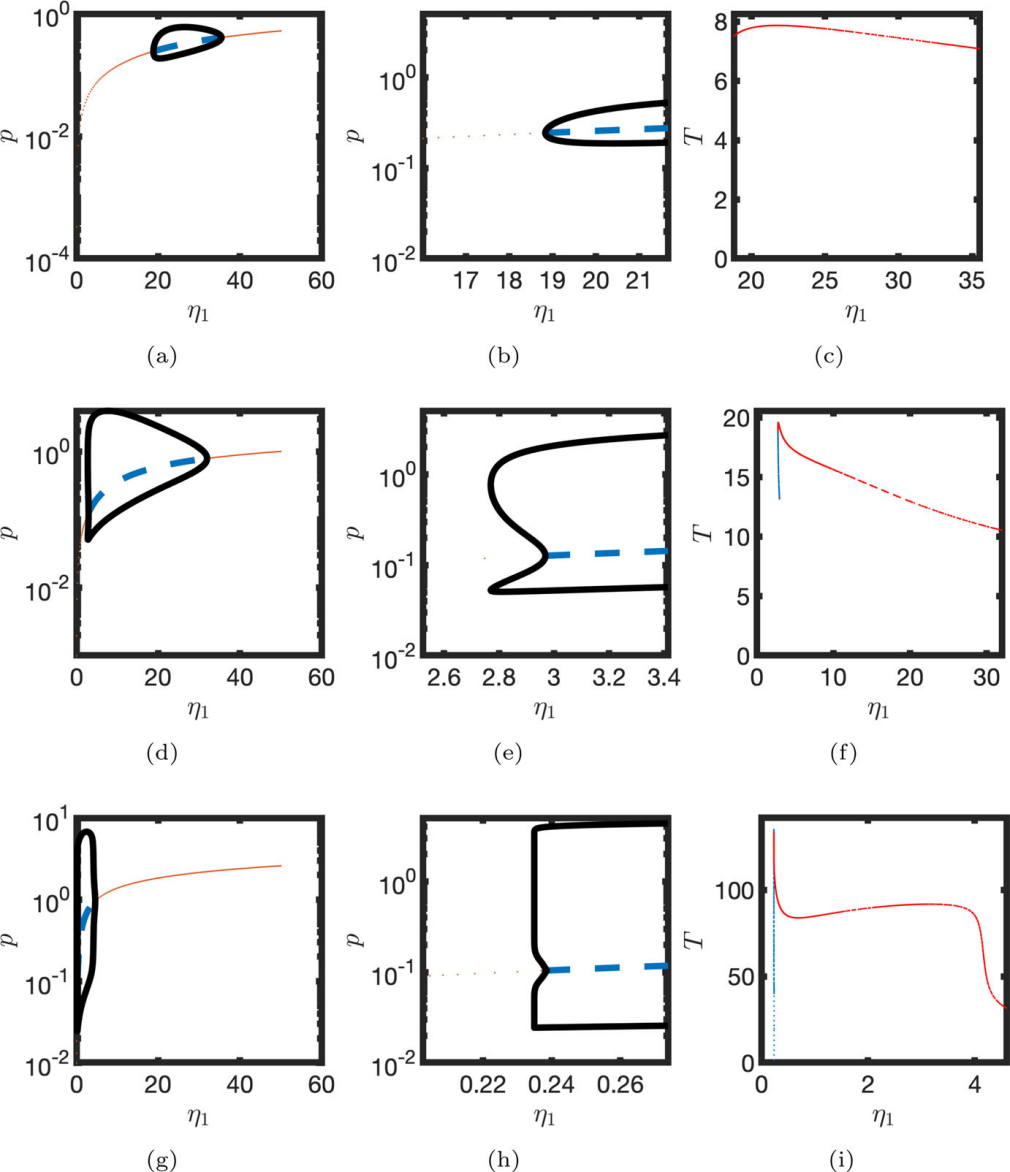
Hopf bifurcations for different values of the parameter $\eta _3$. Top row, $\eta _3=0.6$. Middle row, $\eta _3=0.2$. Bottom row, $\eta _3=0.02$. Left column, steady state levels of protein, *p*, plotted against $\eta _1$ (blue dashed line, unstable; red line, stable). Solid black lines represent maxima/minima of the *p* component of limit cycle solutions. Middle column—inset for left column. Right column—oscillator period is plotted against $\eta _1$. Red markers—stable limit cycle. Blue markers—unstable limit cycle. Parameters as in Table 1 unless otherwise stated (Color figure online)

Numerical continuation also indicates that the classification of the Hopf bifurcation that arises for smaller $\eta _1$ is dependent on the parameter $\eta _3$. For larger $\eta _3$, there are two supercritical Hopf bifurcations. Here the amplitude of oscillations increases close to both bifurcation points (see Fig. 4a–c). However, for smaller $\eta _3$ the Hopf bifurcation is subcritical and one observes the emergence of a saddle node bifurcation of the limit cycle. In this case there is an interval of the parameter $\eta _1$ in which there is an unstable limit cycle, a stable steady state and a stable limit cycle (see Fig. 4d–f). In the limiting case, where both $\eta _1$ and $\eta _3$ are small, the time scale of mRNA production and degradation are relatively long and the system behaves like a relaxation oscillator (see Fig. 4g–i). Notably, the dependence of the oscillator period on the parameter $\eta _1$ is in general not monotonic.

### Period and Amplitude Estimate in the Relaxation Oscillator Limit

**Fig. 5:**
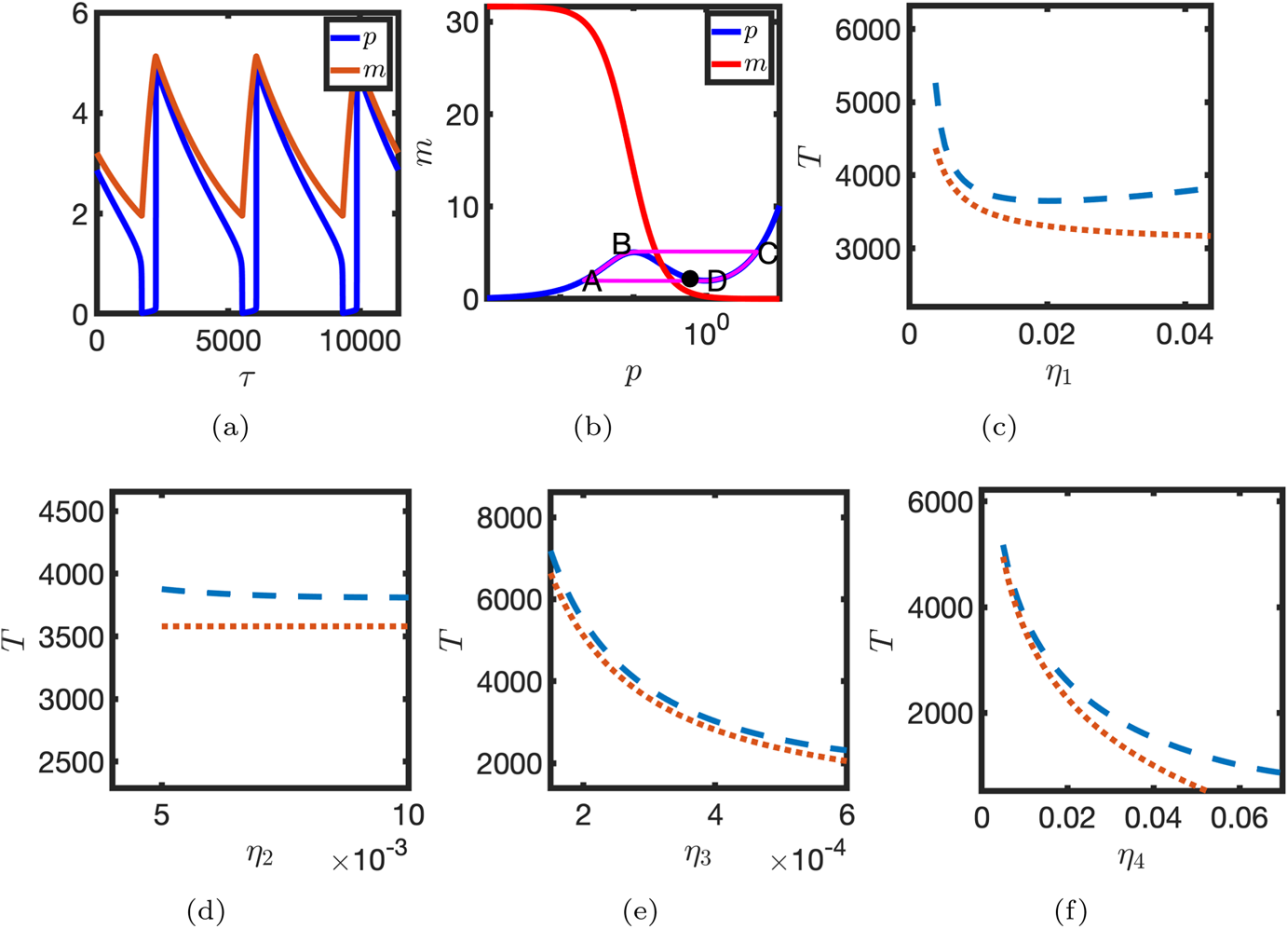
Estimation of the oscillator period in the relaxation oscillator limit. **a** *m* and *p* are plotted against time, $\tau$. **b** Phase plane trajectory. *p* nullcline (blue line). *m* nullcline (red line). Solution trajectory (solid magenta line). **c**–**f** The approximate oscillator period, *T*, is plotted against **c** $\eta _1$, **d** $\eta _2$, **e** $\eta _3$ and **f** $\eta _4$. Numerical estimates (dashed lines) were obtained by solving Eq. (2) numerically. Dotted lines [Eq. (17)]. $\eta _1=0.009$, $\eta _3=0.0003$. Other parameter values as in Table 1 (Color figure online)

Under the assumption that inequalities (14) hold, an estimate for the oscillator period can be derived. Consider the case where the time scale of mRNA transcription and degradation is much longer than that of translation. After applying a fast-slow time scale analysis, where the mRNA is the slow variable, the limit cycle is approximated by a trajectory ABCD (see Fig. 5b) with coordinates

16 $$\begin{aligned} (m_A,p_A)&=(2-2{\eta _4}, 2\eta _4), \quad (m_B,p_B)= \left( \frac{1}{2\sqrt{\eta _4}}(1+{\eta _4}),\sqrt{\eta _4}(1+2\eta _4)\right) , \nonumber \\ (m_C,p_C)&= \left( \frac{1}{2\sqrt{\eta _4}}(1+ \eta _4), \frac{1}{2\sqrt{\eta _4}}(1+ \eta _4) \right) \quad \text {and} \quad (m_D,p_D)=(2-2{\eta _4},1-2\eta _4). \end{aligned}$$

A lower bound for the oscillator period (see Appendix E) is given by

17 $$\begin{aligned} T\sim T_{AB}+T_{CD} = \frac{1}{2 \eta _1\sqrt{\eta _4}}+\frac{1}{\eta _3}\ln \left( \frac{1}{4\sqrt{\eta _4}}\right) . \end{aligned}$$

Thus in the relaxation oscillator limit the period varies inversely with the parameter $\eta _1$. The amplitudes of protein and mRNA oscillation are approximated by

$$\begin{aligned} A_P=p_C-p_A =\frac{1}{2\sqrt{\eta _4}}-2\eta _4\sim \frac{1}{2\sqrt{\eta _4}} \quad \text {and} \quad A_M=m_B-m_A\sim \frac{1}{2\sqrt{\eta _4}}, \end{aligned}$$

respectively. In Fig. 5c–f the derived estimates for the oscillator period are compared with numerical estimates. It is noted that as the oscillator is made less stiff, a correction is needed to Eq. (17) that accounts for time spent close to the local maximum of the *p* nullcline (see Appendix E). In this case the estimate of the oscillator period no longer depends monotonically on the parameter $\eta _1$.

### Dimensional Parameters

#### The Oscillatory Region

Returning to dimensional parameters, the condition $\eta _4<1/9$ (see Appendix A) implies that

$$\begin{aligned} \frac{k_4}{k_3}>8 \quad \text {and} \quad \frac{\alpha }{x_0}>8. \end{aligned}$$

Thus for the *p* nullcline to have two turning points there must be a significant upregulation of the net translation rate and the maximal level of *X* must be much larger than the IC50 for the upregulation of the translation rate. Upon expansion in the small parameter $\eta _4$, condition (13) can be approximated by

18 $$\begin{aligned} \frac{k_2}{k_5}<1-4\sqrt{\frac{X_0}{\alpha }+\frac{k_3}{k_4}}. \end{aligned}$$

These conditions imply the more restrictive bounds

$$\begin{aligned} \frac{\alpha }{X_0}>16 \quad \text {and} \quad \frac{k_4}{k_3}>16. \end{aligned}$$

**Fig. 6:**
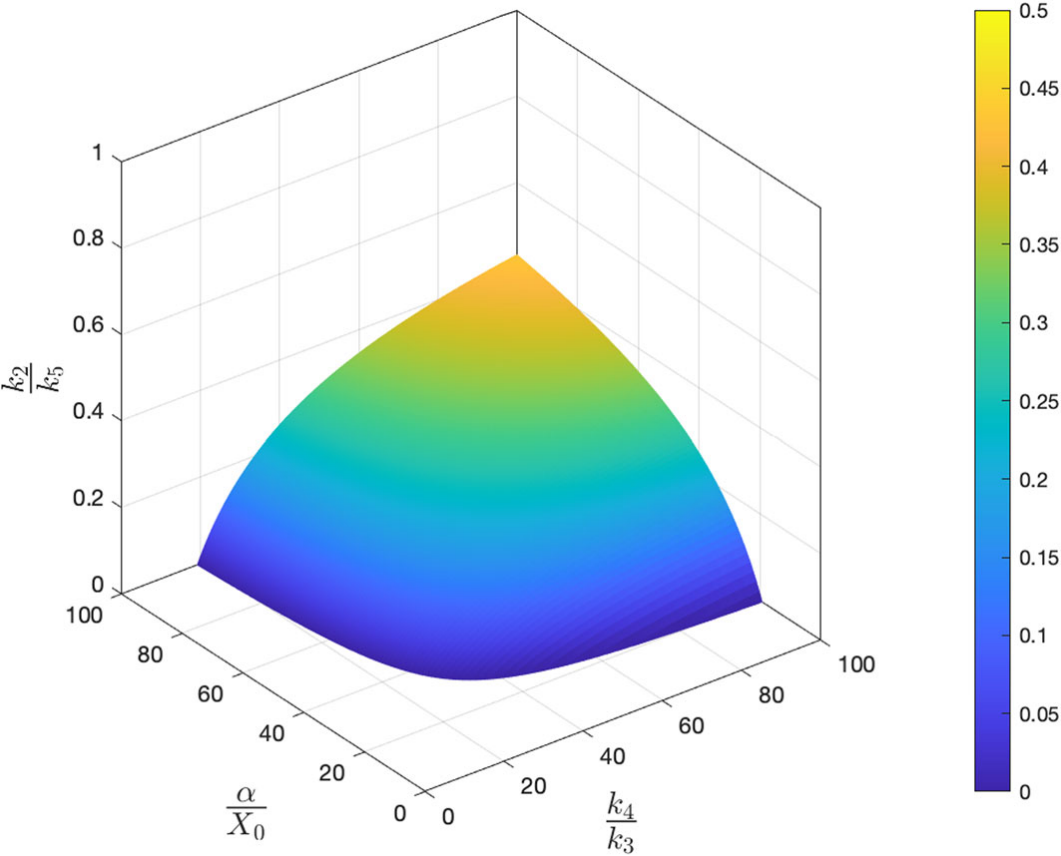
Necessary conditions for instability of the unique steady state. Values of $k_2/k_5$ below which instability is possible are plotted against $k_4/k_3$ and $\alpha /X_0$ [see Eq. (18)] (Color figure online)

A region of parameter space in which oscillations are possible is depicted in Fig. 6. The results imply that an experimental perturbation that independently either: (i) decreases the translation rate ratio; (ii) decreases the steady state level of X; or (iii) decreases mRNA stability relative to protein stability could be sufficient to move the system out of the oscillatory regime. Moreover, mRNA must be more stable the protein in order for oscillatory solutions to be possible.

Upon redimensionalising Eq. (17), an estimate for the oscillator period in the relaxation oscillator limit is given by

$$\begin{aligned} T= \frac{k_5P_0}{2k_1k_4\sqrt{1+\frac{\alpha }{X_0}\frac{k_3}{k_4}}}\frac{\alpha }{X_0} + \frac{1}{k_2}\ln \left( \frac{\sqrt{\frac{k_4}{k_3}}\sqrt{\frac{\alpha }{X_0}}}{4\sqrt{\frac{k_4}{k_3}+\frac{\alpha }{X_0}}}\right) . \end{aligned}$$

Notably, whilst the oscillator period increases linearly with the mRNA half-life ($\ln 2/k_2$), it has an inverse dependence on the protein half life ($\ln 2/k_5$). It is also inversely dependent on the transcription rate, $k_1$, and there is a strong nonlinear dependence on translation rates ($k_3$ and $k_4$). The dimensional protein and mRNA oscillator amplitudes are given at leading order in the relaxation oscillator limit by

$$\begin{aligned} A_P= \frac{P_0}{2}\frac{\alpha }{X_0} \left( \frac{1}{1+\frac{\alpha }{X_0}\frac{k_3}{k_4}}\right) ^{\frac{1}{2}} \quad \text {and} \quad A_M = \frac{P_0}{2}\frac{k_5}{k_4}\frac{\alpha }{X_0} \left( \frac{1}{1+\frac{\alpha }{X_0}\frac{k_3}{k_4}}\right) ^{\frac{1}{2}}, \end{aligned}$$

respectively. Notably, the amplitude of protein and mRNA oscillations are independent of mRNA production and degradation rates. Moreover, the ratio of protein to mRNA amplitudes can be approximated by

$$\begin{aligned} \frac{A_P}{A_M}=\frac{k_4}{k_5}. \end{aligned}$$

## Discussion

A model of the Notch signalling pathway was recently developed in which it was assumed that an intermediate factor that is under the same transcriptional regulation as Hes/Her genes inhibits the translation rate of transcribed mRNA (Murray et al. 2021). Numerical simulations were previously used to explore model behaviour and a number of experimentally testable hypotheses were defined. However, qualitative analysis of the proposed model is required in order to better characterise its behaviours and allow it to be applied in other contexts.

In this study the previous model was nondimensionalised. It was shown that the *p* nullcline had biologically relevant extrema if the parameter $\eta _4$ is sufficiently small. The biological interpretation of this result is that for nontrivial behaviours levels of X must be sufficiently high so as to significantly downregulate the translation rate. In order that model parameters are biologically relevant it was also found that $\eta _2<\eta _4$, i.e. the effective IC50 for transcriptional repression is an order of magnitude smaller than the IC50 for the switch of translation from low to high rates.

After performing a steady state analysis, it was shown that the model possesses a unique steady state for biologically relevant parameter values. Linear stability analysis demonstrated that the unique steady state was linearly stable when the intersection of the nullclines occurs on either the left- or right-most branches of the *p* nullcline. In the case where the steady state arises on the middle branch of the *p* nullcline, it is conditionally stable. If the mRNA is more stable than the protein ($\eta _3<1$) one can always identify an interval of the parameter $\eta _1$ such that the unique steady state is unstable. Upon application of the Poincare Bendixson theorem, there is therefore always a range of $\eta _1$ that yields oscillatory solutions given (provided $\eta _3$, $\eta _4$ and $\eta _2$ are sufficiently small).

Application of numerical continuation confirmed that there is a minimal value of the parameter $\eta _3$ below which oscillatory solutions can be found. Moreover, as $\eta _1$ increases from below there is Hopf bifurcation that is either subcritical or supercritical. Notably, a previous study of isolated zebrafish PSM cells has postulated a Stuart Landau model which has a supercritical Hopf bifurcation, behaviour that is consistent with the proposed model (Webb et al. 2016).

In the limit where the rate constants associated with mRNA ($\eta _1$ and $\eta _3$) are chosen to be relatively small, the model behaves like a relaxation oscillator. In this case the period is approximated by assuming that the trajectory is in quasi-equilibrium on the left- and right branches of the *p* nullcline. Close to the local maximum of the *p* nullcline dynamics are relatively slow and an extra term must be accounted for that describes the time taken for protein levels to increase sufficiently so as to upregulate the translation rate.

The dimensionless equations (2) have previously been proposed as an illustrative model that describes how coupled positive and negative feedback loops give rise to ‘frustrated bistability’ (Krishna et al. 2009). The analysis performed heres generalises the work of Krishna et al. (2009) by considering dependence of model behaviour on the parameter $\eta _4$ as well as deriving explicit formulae for the oscillator period and bounds for the domain of oscillatory solutions. Moreover, the derivation of the model in this study differs from that of Krishna et al. (2009); here we consider regulation of the transcriptional and translational products of a single gene whilst Krishna et al. (2009) consider a model for protein-protein interaction.

The role of Notch signalling in regulating the period of the segmentation clock oscillator appear to be species dependent. In the zebrafish embryo it has been shown that levels of Notch signalling are anticorrelated with the oscillator period. When Notch signalling is increased via overexpression of Delta ligand the period decreases (Liao et al. 2016). Moreover, when levels of Notch signalling are reduced via gamma secretase treatment the period of the segmentation clock increases (Herrgen et al. 2010). In contrast, when mouse and chicken embryos are pharmaceutically treated with compounds that increase levels of the Notch intracellular domain, the period of the segmentation clock is increased (Wiedermann et al. 2015). In the proposed model intercellular coupling is not explicitly accounted for. Rather, the parameter $k_1$ (and hence $\eta _1$) can act as a proxy for levels of Notch signalling (assuming that levels of NICD regulate the maximal transcription rate). In this study it has been shown that the oscillator period can either increase or decrease with $\eta _1$. Thus the proposed model supports the hypothesis that species-specific differences in rate constants could explain the contrasting observation of the dependence of oscillator period on levels of Notch signalling.

It is notable that oscillatory solutions of the model are permitted only if mRNA is more stable than protein. Whilst in mouse fibroblasts mRNAs have been measured to be on average approximately fives times less stable than the protein that they encode, this is not true for approximately 10% of mRNAs (Schwanhäusser et al. 2011). Gene ontology analysis associates genes that encode relatively stable mRNAs with biological processes such as tissue morphogenesis, cell proliferation, phosphorylation and positive regulation of signal transduction (Schwanhäusser et al. 2011). Moreover, direct measurement of Hes1 mRNA and protein in mouse PSM tissue yielded half lives of 24.1 and 22.3 min, respectively (Hirata et al. 2002). Additionally, in zebrafish the half life of Her7 protein has been measured to be to 3.5 min at 24$^{\circ }$ and it has been inferred using simulations that the mRNA half life is between 2 and 6 min (Ay et al. 2013). Together, these measurements suggest that Hes1/Her7 genes encode mRNA and proteins that have similar half lives.

The relaxation oscillator analysis yields a number of experimentally testable predictions. For example, a decrease in the parameter $$k_5$$ (i.e. more stable protein) would result in a smaller oscillatory period and an increase in the amplitude of protein oscillation relative to that of the mRNA. In contrast, a decrease in parameter $$k_2 $$ (i.e. making the mRNA more stable) would result in a larger period of oscillation but with an unchanged oscillation amplitude. These predictions allow for the proposed model to be distinguished from delayed negative feedback models where the oscillator period is predicted to increase linearly with both the protein and mRNA half lives Lewis (2003).

In this study a qualitative analysis has been performed on a model of a gene regulatory network in which translation as well as transcription rates are regulated by the product of a pathway. The main finding is that oscillatory solutions are possible only when: the regulation of translation rate is sufficiently large and mRNA is sufficiently more stable than protein. The qualitative analysis allows for the previous model to be applied in different biological contexts.

## Figures and Tables

**Fig. 7:**
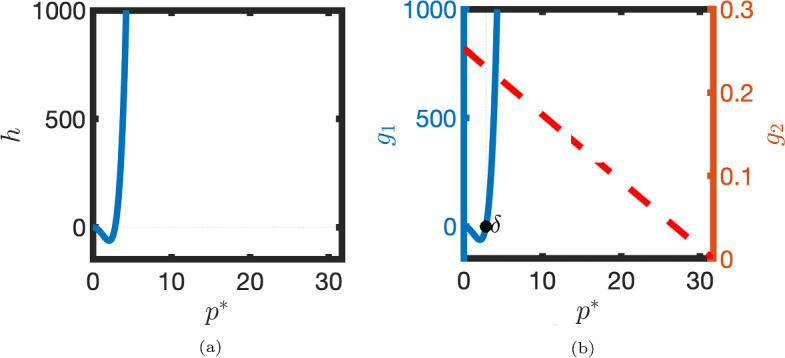
**a** *h* [Eq. (6)] is plotted against $p^*$. **b** $g_1$ [Eq. (23), solid blue line] and $g_2$ [Eq. (24), dashed red line] are plotted against $p^*$ (Color figure online)

**Fig. 8:**
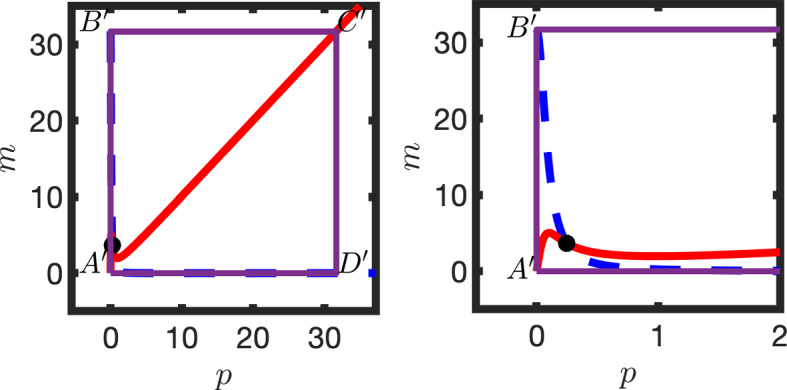
**a** A confined set is depicted in the *mp* phase plane. *p* nullcline (solid red line) *m* nullcline (dashed blue line). The confined set is given by $A'B'C'D'A'$. **b** Inset for **a** (Color figure online)

**Fig. 9:**
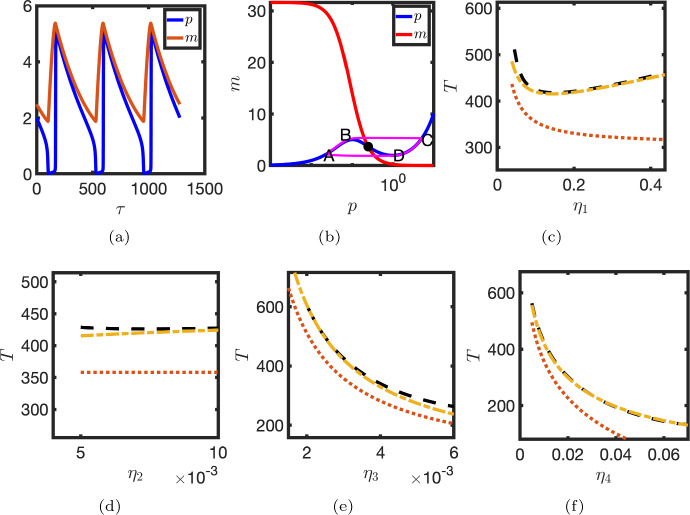
Estimation of the oscillator period in the relaxation oscillator limit. $\eta _1=0.09$, $\eta _3=0.003$. (c-f) Dot-dashed line [Eq. (31)]. Other details as in Fig. 5 (Color figure online)

## Appendix A Nullclines

For small *p* the asymptote of the *p* nullcline is given by the line $m= p/\eta _4$ whilst for large *p* it is given by the line $m=p$. To identify turning points of the *p* nullcline $m_2(p)$, Eq. (4) is differentiated with respect to *p* and the equation

19 $$\begin{aligned} \frac{\textrm{d}{\bar{m}}_2}{\textrm{d}p} =\frac{p^4+p^2(3\eta _4-1)+\eta _4}{(\eta _4+p^2)^2}=0, \end{aligned}$$

is solved. Turning points thus occur at

20 $$\begin{aligned} p_c=\pm \left( \frac{1-3\eta _4 \pm \sqrt{(1-9\eta _4)(1-\eta _4)}}{2} \right) ^{\frac{1}{2}}. \end{aligned}$$

A necessary condition for unique real turning points is that

$$\begin{aligned} (1-9\eta _4)(1-\eta _4)> 0, \end{aligned}$$

an inequality that is satisfied in the intervals

$$\begin{aligned} \eta _4< \frac{1}{9} \quad \text {and} \quad \eta _4> 1. \end{aligned}$$

For $p_c\in \Re ^+$, a further requirement is that

$$\begin{aligned} \eta _4<\frac{1}{3}. \end{aligned}$$

Hence there are two unique real positive turning points if

$$\begin{aligned} \eta _4<\frac{1}{9}. \end{aligned}$$

### A.1 An Approximation for the Turning Points of the *p* Nullcline

Given that $\eta _4<1/9$ then, upon applying the binomial expansion to Eq. (20), the local maximum and minimum are approximated by

$$\begin{aligned} (p_1,m_1)=\left( {\sqrt{\eta _4}}(1+2\eta _4+O(\eta _4^2)),\frac{1}{2\sqrt{\eta _4}}\left( 1+\eta _4+O(\eta _4^2)\right) \right) , \end{aligned}$$

and

$$\begin{aligned} (p_2,m_2)=(1-2{\eta _4}+O(\eta _4^2),2-2{\eta _4}+O(\eta _4^2)), \end{aligned}$$

respectively. Note that $(p_1,m_1)$ is a local maximum and $(p_2,m_2)$ a local minimum of the *p* nullcline.

### A.2 Parameter Constraints in the Case of a Non Monotonic *p* Nullcline

Consider the definitions of $\eta _2$ and $\eta _4$ given in Eq. (3). The parameter ratio $\frac{k_4}{k_3}$ can be expressed in terms of dimensionless parameters in the form

21 $$\begin{aligned} \frac{k_4}{k_3}=\frac{1-\eta _4}{\eta _4-\eta _2}. \end{aligned}$$

Hence in the case $\eta _4<1$, the assumption that the parameters $k_3$ and $k_4$ are positive implies that

$$\begin{aligned} \eta _2<\eta _4. \end{aligned}$$

Moreover, the constraints $\eta _2<\eta _4<1/9$ imply that

$$\begin{aligned} \frac{k_4}{k_3}>8. \end{aligned}$$

Hence the translation rate that is nonlinearly regulated must be significantly larger than the background rate $k_3$ in order for the *p* nullcline to be non-monotonic. Furthermore, using the definition of $\eta _2$ in Eq. (3) together with the constraint $\eta _2<\eta _4<1/9$ implies that

$$\begin{aligned} \frac{\alpha }{X_0}>8. \end{aligned}$$

Recall that the quasi steady state approximation for *X* yields

$$\begin{aligned} X= \frac{\frac{\alpha }{X_0}}{1+\left( \frac{P}{P_0}\right) ^2}. \end{aligned}$$

Hence for the *p* nullcline to be non-monotonic, *X* must be sufficiently large in magnitude such that it can have a a strong effect on the net translation rate.

## Appendix B Steady State Analysis

Suppose that $(p^*,m^*)$ is a steady state solution of equations (2). $p^*$ satisfies the fifth order polynomial

22 $$\begin{aligned} h(p^*)={p^*}^5+{p^*}^3(\eta _2+1)-{p^*}^2 \frac{\eta _1\eta _2}{\eta _3}+\eta _2{p^*}-\frac{\eta _1\eta _2\eta _4}{\eta _3}=0. \end{aligned}$$

The *m* nullcline [Eq. (5)] is bounded above by $m=\eta _1/\eta _3$. Hence $m^*<\eta _1/\eta _3$.

The *p* nullcline [Eq. (4)] is bounded below by the line $m=p$ $\forall p>0$. Hence $m^*>p^*$ and therefore

$$\begin{aligned} p^*\in [0,\eta _1/\eta _3]. \end{aligned}$$

Consider the steady state Eq. (22). Define

23 $$\begin{aligned} g_1({p^*})={p^*}^2\left( {p^*}^3+{p^*}(1+\eta _2)-\frac{\eta _1\eta _2}{\eta _3}\right) , \end{aligned}$$

and

24 $$\begin{aligned} g_2({p^*})=\eta _2\left( \frac{\eta _1\eta _4}{\eta _3}-{p^*}\right) , \end{aligned}$$

such that Eq. (6) can be expressed as $g_1(p^*)=g_2(p^*)$.

The function $g_2({p^*})$ is linear in $p^*$ with the intercept at

$$\begin{aligned} g_2(0)=\frac{\eta _2\eta _1\eta _4}{\eta _3}, \end{aligned}$$

and root at

$$\begin{aligned} {p^*}=\frac{\eta _1\eta _4}{\eta _3}. \end{aligned}$$

$g_1({p^*})$ is a fifth order polynomial. Note that

$$\begin{aligned} g_1(0)=0, \quad g_1'(0)=0 \quad \text {and} \quad g_1''(0)<0. \end{aligned}$$

As the leading order term at $p^*=0$ is negative and $g_1\rightarrow \infty$ as $p^*\rightarrow \infty$, $g_1$ has at least one real positive root. Moreover, applying Descartes’ rule of signs, $g_1$ has at most one real positive root. Hence $g_1$ has a unique real positive root at $p^*=\delta \in [0, \eta _1/\eta _3]$.

Turning points of $g_1$ satisfy

$$\begin{aligned} g_1'=5{p^*}{^4}+3{p^*}^2 (1+\eta _2)-2p^*\frac{\eta _1\eta _2}{\eta _3}=0 \end{aligned}$$

Application of Descartes rule of signs implies that $g_1$ has at most one turning point for $p^*>0$. Hence in the interval $p^*\in [0,\delta ]$, $g_1<0$ and $g_1$ has a unique turning point (see Fig. 7).

Consider the interval $[0,\delta ]$. As $g_1<0$ and $g_2>0$, the steady state solution $p^*$ does not lie in the interval $[0,\delta ]$.

Now consider the interval $\big [\delta ,\frac{\eta _1}{\eta _3}\big ]$. At $\eta _1/\eta _3$

$$\begin{aligned} g_1\left( \frac{\eta _1}{\eta _3}\right) >0, \end{aligned}$$

and

$$\begin{aligned} g_2\left( \frac{\eta _1}{\eta _3}\right) =0. \end{aligned}$$

Hence

$$\begin{aligned} g_1\left( \frac{\eta _1}{\eta _3}\right) >g_2\left( \frac{\eta _1}{\eta _3}\right) . \end{aligned}$$

At $p^*=\delta$, $g_1(\delta )=0$ and $g_2(\delta )>0$. Thus

$$\begin{aligned} g_1\left( \delta \right) <g_2\left( \delta \right) . \end{aligned}$$

As in the interval $\big [\delta ,\frac{\eta _1}{\eta _3}\big ]$

$$\begin{aligned} g_2'({p^*})<0 \end{aligned}$$

and

$$\begin{aligned} g_1'({p^*})>0, \end{aligned}$$

there is one intersection $\gamma \in \big [\delta ,\frac{\eta _1}{\eta _3}\big ]$. Hence Eq. (22) has a unique steady state in $[\delta ,\eta _1/\eta _3]$.

### B.1 $p^*$ is a Monotonically Increasing Function of $\eta _1$

Differentiating Eq. (6) with respect to $\eta _1$ yields

$$\begin{aligned} \frac{dp^*}{d\eta _1}= \frac{\frac{\eta _2}{\eta _3}({p^*}^2+\eta _4)}{5{p^*}^4+3{p^*}^2(1+\eta _2)-2p^*\frac{\eta _1\eta _2}{\eta _3}+\eta _2}. \end{aligned}$$

As the numerator is positive, the derivative is positive if the condition

$$\begin{aligned} 5{p^*}^4+3{p^*}^2(1+\eta _2)-2p^*\frac{\eta _1\eta _2}{\eta _3}+\eta _2>0 \end{aligned}$$

holds for any $p^*$ that is a solution of Eq. (22). This inequality holds in the case of a unique bounded solution to the steady state problem.

In the case where $\eta _2<1/9$ this inequality is satisfied for $p^*>\frac{2}{27}\frac{\eta _1}{\eta _3}$. Hence the inequality is satisfied for all $p^*\in [0,\eta _1/\eta _3]$. Hence $p^*$ is an increasing function of the parameter $\eta _1$.

## Appendix C Linear stability analysis

Positivity of the determinant of the Jacobian matrix requires

$$\begin{aligned} -\eta _3(m^*f'(p^*)-1)+\frac{2p \eta _1}{\eta _2}\frac{f(p^*)}{(1+\frac{p^*}{\eta _2})^2}>0, \end{aligned}$$

where

$$\begin{aligned} f(p^*)=\frac{\eta _4+{p^*}^2}{1+{p^*}^2}. \end{aligned}$$

Substituting for

$$\begin{aligned} m^*=\frac{\frac{\eta _1}{\eta _3}}{\left( 1+\frac{{p^*}^2}{\eta _2}\right) }, \end{aligned}$$

yields, after rearrangement and simplification,

$$\begin{aligned} {p^*}^4 + \eta _4 {p^*}^2 + (\eta _4-\eta _2)>0. \end{aligned}$$

Noting that

$$\begin{aligned} \eta _2< \eta _4, \end{aligned}$$

the inequality is therefore satisfied $\forall p>0$. Hence the Jacobian determinant is positive definite.

## Appendix D Poincare Bendixson

A confined set of Eq. (2) is given by $\mathrm{A'B'D'C'A'}$ (see Fig. 8). Let A’ represent the origin and $\textrm{B}^{\prime }$ represent the point $(0,\eta _1/\eta _3)$. The outward unit normal on A’B’ is ${{\textbf {n}}}_{A'B'}=[-1,0 ]$. On this line segment

$$\begin{aligned} {{\textbf {n}}}_{A'B'}\cdot \left[ \frac{dp}{d\tau }, \frac{dm}{d\tau }\right] =-\frac{dp}{d\tau }< 0. \end{aligned}$$

Let $\mathrm{C'}$ represent the point $(p^{\dagger },\eta _1/\eta _3)$ such that

$$\begin{aligned} \frac{p^{\dagger }(1+{p^{\dagger }}^2)}{\eta _4+{p^{\dagger }}^2}=\frac{\eta _1}{\eta _3}. \end{aligned}$$

$p^{\dagger }$ is uniquely defined so long as $\eta _1/\eta _3>m_1$. The outward unit normal on $\textrm{B}^{\prime }\textrm{C}^{\prime }$ is ${{\textbf {n}}}_{B'C'}=[0,1]$. On $\mathrm{B'C'}$

$$\begin{aligned} {{\textbf {n}}}_{B'C'}\cdot \left[ \frac{dp}{d\tau }, \frac{dm}{d\tau }\right] =-\frac{dm}{d\tau }< 0. \end{aligned}$$

Let $\mathrm{D'}$ represent $(p^{\dagger },0)$. The outward unit normal on C’D’ is ${{\textbf {n}}}_{C'D'}=[1,0]$. On C’D’

$$\begin{aligned} {{\textbf {n}}}_{C'D'}\cdot \left[ \frac{dp}{d\tau }, \frac{dm}{d\tau }\right] =-\frac{dp}{d\tau }< 0. \end{aligned}$$

Finally, on D’A’ the outward unit normal is ${\textbf{n}}_{D'A'}=[0,-1]$. On D’A’

$$\begin{aligned} {{\textbf {n}}}_{D'A'}\cdot \left[ \frac{dp}{d\tau }, \frac{dm}{d\tau }\right] =-\frac{dm}{d\tau }<0. \end{aligned}$$

Hence $\mathrm{A'B'C'D'A'}$ defines a confined set. Therefore, by the Poincare Bendixson theorem, when the unique steady state is linearly unstable, the solution is a stable limit cycle.

## Appendix E Period estimate

Let

$$\begin{aligned} \eta _1=\epsilon {\hat{\eta }}_1 \quad \text {and} \quad \eta _3=\epsilon {\hat{\eta }}_3, \end{aligned}$$

where

$$\begin{aligned} \epsilon \ll \sqrt{\eta _4}<\frac{1}{9}. \end{aligned}$$

Equations (2) transform to

25 $$\begin{aligned} \frac{\textrm{d} m}{\textrm{d}\tau }&=\epsilon \left( \frac{{\hat{\eta }}_1}{1+\frac{p^2}{\eta _2}} - {\hat{\eta }}_3 m\right) , \nonumber \\ \frac{\textrm{d} p}{\textrm{d}\tau }&=m\frac{(\eta _4+ p^2)}{1+p^2}-p, \end{aligned}$$

and an oscillatory solution can be approximated using a slow-fast timescale analysis.

Consider the trajectory ABCDA with coordinates

26 $$\begin{aligned} (m_A,p_A)&=(2-2{\eta _4}, 2\eta _4), \quad (m_B,p_B)= \left( \frac{1}{2\sqrt{\eta _4}}(1+{\eta _4}),\sqrt{\eta _4}(1+2\eta _4)\right) , \nonumber \\ (m_C,p_C)&= \left( \frac{1}{2\sqrt{\eta _4}}(1+ \eta _4), \frac{1}{2\sqrt{\eta _4}}(1+ \eta _4) \right) \quad \text {and} \quad (m_D,p_D)=(2-2{\eta _4},1-2\eta _4). \end{aligned}$$

On the segment *AB* *p* is assumed to be in quasi-equilibrium, i.e.

$$\begin{aligned} m=\frac{p(1+p^2)}{\eta _4+ p^2} \end{aligned}$$

and the ODE

$$\begin{aligned} \frac{\textrm{d}p}{\textrm{d}\tau }=\frac{dp}{dm}\frac{dm}{d\tau }=\epsilon \frac{(\eta _4+p^2)^2}{p^4+p^2(3\eta _4-1)+\eta _4}\left( \frac{{\hat{\eta }}_1}{1+\frac{p^2}{\eta _2}}-{\hat{\eta }}_3\frac{p(1+p^2)}{\eta _4+p^2}\right) . \end{aligned}$$

is integrated from $p_A$ to $p_B$. After applying separation of variables the time spent on the segment AB is

27 $$\begin{aligned} T_{AB}=\frac{1}{\epsilon } \int _{p_A}^{p_B} \frac{\frac{dm}{dp}}{\frac{dm}{d\tau }} dp = \frac{1}{\epsilon } \int _{p_A}^{p_B} \frac{p^4+p^2(3\eta _4-1)+\eta _4}{(\eta _4+p^2)^2\left( \frac{{\hat{\eta }}_1}{1+\frac{p^2}{{\eta }_2}}-{\hat{\eta }}_3\frac{p(1+p^2)}{\eta _4+p^2}\right) }dp. \end{aligned}$$

The integral in Eq. (27) is approximated as follows. As the term

$$\begin{aligned} g(p)=\left( \frac{{\hat{\eta }}_1}{1+\frac{p^2}{{\eta }_2}}-{\hat{\eta }}_3\frac{p(1+p^2)}{\eta _4+p^2}\right) \end{aligned}$$

is a decreasing function of *p* for $p\in [p_A,p_B]$ it is bounded in the interval [$g(p_B)$, $g(p_A)$].

Hence

28 $$\begin{aligned} T_{AB}&\in \left[ \frac{1}{g(p_A)},\frac{1}{g(p_B)}\right] \frac{1}{\epsilon } \int _{m_A}^{m_B} dm \nonumber \\ \implies T_{AB}&\in \frac{1}{\epsilon } \left[ \frac{1}{g(p_A)},\frac{1}{g(p_B)}\right] \left( \frac{1}{2\sqrt{\eta _4}}-2+\frac{\sqrt{\eta _4}}{2}+2\eta _4+O(\eta _4^{\frac{3}{2}})\right) . \end{aligned}$$

At point B

$$\begin{aligned} \frac{dm}{d\tau }\sim O(\epsilon ) \quad \text {and} \quad \frac{dp}{d\tau }\sim O(\sqrt{\eta _4}). \end{aligned}$$

Given that $\epsilon \ll \sqrt{\eta _4}$, on the segment BC *m* is a slow variable, approximated by

$$\begin{aligned} m\sim m_B, \end{aligned}$$

and *p* rapidly increases until the trajectory reaches the point C on the right branch of the *p* nullcline.

On the segment CD, *p* is assumed to be in quasi-steady state. As $p>1\gg \eta _2$, the *m* dynamics are approximated by

$$\begin{aligned} \frac{\textrm{d}m}{\textrm{d}\tau }=-\eta _3m. \end{aligned}$$

Thus the time spent on the segment CD is

$$\begin{aligned} T_{CD}=\frac{1}{\epsilon {\hat{\eta }}_3}\ln \left( \frac{m_C}{m_D}\right) . \end{aligned}$$

On the segment DA, dynamics are fast. *m* is is approximated by

$$\begin{aligned} m=M_D \end{aligned}$$

and

$$\begin{aligned} \frac{\textrm{d}p}{\textrm{d}\tau }=O(1). \end{aligned}$$

Thus the period is approximately given by

29 $$\begin{aligned} T= T_{AB}+T_{CD}. \end{aligned}$$

Considering the upper bound $g=g(p_A)$ yields the estimate

$$\begin{aligned} T_{AB}=\frac{1}{\epsilon }\left( \frac{1}{g(p_A)} \left( \frac{1}{2\sqrt{\eta _4}}-2+\frac{\sqrt{\eta _4}}{2}+2\eta _4+O(\eta _4^{\frac{3}{2}})\right) \right) . \end{aligned}$$

Hence the period is approximated by

30 $$\begin{aligned} T=\frac{1}{\epsilon }\left( \frac{1}{g(p_A)} \left( \frac{1}{2\sqrt{\eta _4}}-2+\frac{\sqrt{\eta _4}}{2}+2\eta _4+O(\eta _4^{\frac{3}{2}})\right) +\frac{1}{{\hat{\eta }}_3}\ln \left( \frac{1}{4\sqrt{\eta _4}}\frac{1+ \eta _4}{(1-\eta _4)}\right) \right) . \end{aligned}$$

Finally, it is noted that $g<\eta _1$. For simplicity *g* is represented by the upper bound $\eta _1$. Considering leading order in $\eta _4$ yields Eq. (17).

At the local maximum of the *p* nullcline $dp/d\tau$ is $O(\sqrt{\eta _4})$. Hence for $\epsilon \sim \sqrt{\eta _4}$ the fast-slow analysis will become inaccurate. The time for *p* to increase from the local maximum of the *p* nullcline ($p=\sqrt{\eta _4}$) to the IC50 for the translation switch is approximately

$$\begin{aligned} T_{BB'}=\frac{1}{2\sqrt{\eta _4}}-1 \end{aligned}$$

Linearising about the local maximum of the *p* nullcline, *m* increases by amount

$$\begin{aligned} \Delta m = \epsilon \left( \frac{{\hat{\eta }}_1}{1+\frac{\eta _4}{\eta _2}}-{\hat{\eta }}_3\frac{1}{2\sqrt{\eta _4}}\right) T_{BB'} \end{aligned}$$

in time $T_{BB'}$. On the descent this increase in *m* requires an additional time

$$\begin{aligned} T_{B'C}=\frac{1}{\eta _3}\ln \left( 1+\frac{\Delta m}{m_C}\right) . \end{aligned}$$

Thus the period is approximately given by

31 $$\begin{aligned} T= T_{AB}+T_{BB'} +T_{B'C}+T_{CD}. \end{aligned}$$

In Fig. 9 the derived estimates for the oscillator period are compared with numerical estimates. In this case $\eta _1 \sim O(\sqrt{\eta _4})$. Note that the dependence of oscillator period on $\eta _1$ is no longer monotonic. For small $\eta _1$ the period increases as $\eta _1$ decreases as a result of the progression of the solution along AB (transcription is a limiting step). However, for larger $\eta _1$ the period increases with $\eta _1$. This effect is due to the overshoot at the local maximum of the *p* nullcline.
